# Emerging Roles and Landscape of Translating mRNAs in Plants

**DOI:** 10.3389/fpls.2017.01443

**Published:** 2017-09-01

**Authors:** Gaurav Sablok, Jonathan J. Powell, Kemal Kazan

**Affiliations:** ^1^Finnish Museum of Natural History Helsinki, Finland; ^2^Department of Biosciences, Viikki Plant Science Center, University of Helsinki Helsinki, Finland; ^3^Commonwealth Scientific and Industrial Research Organization Agriculture, St. Lucia QLD, Australia; ^4^Queensland Alliance for Agriculture and Food Innovation, University of Queensland, St. Lucia QLD, Australia

**Keywords:** mRNA, translational regulation, ribosomal associations, stress, development

## Abstract

Plants use a wide range of mechanisms to adapt to different environmental stresses. One of the earliest responses displayed under stress is rapid alterations in stress responsive gene expression that has been extensively analyzed through expression profiling such as microarrays and RNA-sequencing. Recently, expression profiling has been complemented with proteome analyses to establish a link between transcriptional and the corresponding translational changes. However, proteome profiling approaches have their own technical limitations. More recently, ribosome-associated mRNA profiling has emerged as an alternative and a robust way of identifying translating mRNAs, which are a set of mRNAs associated with ribosomes and more likely to contribute to proteome abundance. In this article, we briefly review recent studies that examined the processes affecting the abundance of translating mRNAs, their regulation during plant development and tolerance to stress conditions and plant factors affecting the selection of translating mRNA pools. This review also highlights recent findings revealing differential roles of alternatively spliced mRNAs and their translational control during stress adaptation. Overall, better understanding of processes involved in the regulation of translating mRNAs has obvious implications for improvement of stress tolerance in plants.

## Introduction

Plant ‘omics’ research is currently focusing on at least two important fronts that can have major implications for crop breeding: (1) sequencing and re-sequencing of plant genomes with phylogenetic or agronomic importance and (2) comparative and functional genomic approaches for identifying genes with important roles in plant development and stress adaptation. Such genes can be edited using the editing technology such as CRISPR-Cas9 to develop crops with high value traits (Chang et al., 2016; Zhou et al., 2016). Aforementioned approaches are also components of ‘systems biology,’ which broadly refers to a common framework to understand the functional component of plant genomes and their subsequent adaptation to changing climates (Cramer et al., 2011). Recently, high throughput transcriptome profiling approaches such as RNA-seq have revolutionized the discovery and functional characterization of genes associated with agronomically important traits. In addition, several traits of agronomic importance and recently various forms of quantitative trait loci (QTL) such as expression QTL (e-QTL), *cis*- and *trans*-QTL have been mapped for functional crop improvement (Druka et al., 2010; Wang et al., 2014). These approaches have been widely applied across a variety of stress conditions (Hirayama and Shinozaki, 2010; Debnath et al., 2011) to understand the regulatory role of stress responsive genes and associated transcription factors (Fu et al., 2016).

In addition, recently emerging co-expression analyses (Serin et al., 2016) and network modeling approaches have been widely used to identify key networks or modules and certain transcription factors that modulate these networks in those regulatory modules (Serin et al., 2016). Although transcriptome profiling methods have been informative, they do not necessarily provide a thorough understanding of whether transcriptional changes observed under a condition actually mirror the abundance of mRNAs (translating mRNAs) associated with ribosomes. Indeed, ribosome profiling approaches, which profiles mRNAs fragments associated with ribosomes provides a direct estimate of mRNAs to be translated into proteins. A thorough understanding of this process will enable us to focus only on those mRNAs bound to ribosomes for functional analyses of gene function. Emerging evidence from recent studies have suggested the discordance between transcriptome and proteome can even be greater during stress responses. Indeed, translating mRNAs are reduced by ∼50% under heat stress as only those mRNAs encoding proteins mainly involved in translation and stress responses, are enriched for binding to ribosomes, suggesting that they are selectively translated and demonstrates a level of selective enrichment of certain mRNAs during stress (Yángüez et al., 2013). Similarly, 77% decrease in the pool of translating mRNAs was observed under hypoxia stress indicating that the selective enrichment of hypoxia specific genes in the translating mRNA pool (Branco-Price et al., 2005). Taking into account, the collective information, we can infer the swathing information about the translational control of plant under stress and development conditions using the translating mRNAs as an index of measure. In this review, we highlight the role of ribosome profiling in identifying translating mRNAs involved in stress responses and plant development.

## Profiling Approaches for Translating mRNAs

Proteomic-based approaches have been used to establish a correlation between observed fluctuations in transcript expression and the actual peptide abundance during plant development (Galland et al., 2014). However, proteomic approaches are laborious and expensive and preparation of samples and quantification of proteome using techniques such as 1-dimensional (1-D) or 2-dimensional (2-D) or 2D-coupled with iso-electric focusing (2D-IEF) followed by peptide sequencing also requires specialized technical expertise and provide limited resolution of spatio-temporal resolution of translated mRNAs. Another limitation of proteome-based approaches is the identification of an algorithm of sequenced peptides, which mainly relies on BLAST searches against the proteome of the corresponding species or against previously annotated or un-annotated proteins. Recently, ribosomal foot-printing (Ingolia et al., 2009, 2012, 2014), which sequences ribosome-protected mRNA fragments, has been applied to identify mRNAs bound to ribosomes. Additionally, the genome-wide profiling of ribosome-protected mRNA fragments has highlighted the role of 5′ and 3′- regulatory regions and the presence of sequence motifs which could accelerate the initiation of translating mRNAs (Bai et al., 2016).

**FIGURE 1:**
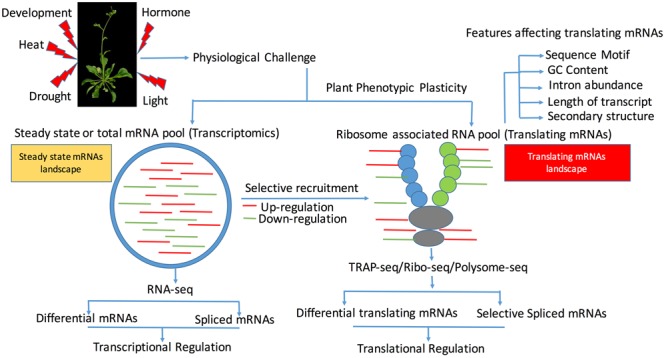
Schematic view of transcriptional (steady state mRNAs) and translating (mRNA bounds to ribosomes) mRNAs in plants across abiotic stress and development. Induction of abiotic stress or changes in stages of development requires specific set of the transcriptional mRNAs, which is represented by the steady state mRNAs and translationally active mRNAs as represented by the ribosome bound mRNAs. Differential regulation of these steady state mRNAs and translating mRNAs play a major role in defining the phenotypic plasticity of the plant to a stress environment. Translating mRNAs represent a subset of pool of mRNAs, which are bound to ribosomes and can be identified using either intact ribosomal immunoprecipitation or TRAP-Seq, translating ribosome affinity purification (TRAP), which involves the RNA-sequencing of the purified ribosomes. To reduce the contamination of the messenger ribonucleoproteins (mRNPs), FLAG-tagged ribosomal protein L18 (RPL18) is used.

To isolate translating mRNAs, either intact ribosomes or immunopreciptated ribosomes enriched for polysome-associated mRNAs have been used (Zanetti et al., 2005; Reynoso et al., 2015; Zhao et al., 2017). Translational abundance is then analyzed using ribosome-profiling methods such as translating ribosome affinity purification (TRAP) followed by RNA sequencing (TRAP-seq) (Zanetti et al., 2005). The use of FLAG-tagged ribosomal protein L18 (RPL18) in this method gives an intricate view of functional ribosomes by reducing the contamination of messenger ribonucleoproteins (mRNPs) (Juntawong et al., 2013; Zhao et al., 2017). **Figure 1** represents a summary view of applications of these approaches to study stress conditions and developmental patterns. These approaches have been undertaken in both model and non-model species with model species benefiting from the availability of genome-based mapping methods, which can reveal the translational efficiency as a measure of the ribosomal scores (Guttman et al., 2013). Recently, ribosomal scores have been used to measure the translational efficiency of sense and antisense transcripts in maize (*Zea mays*) under drought stress (Xu et al., 2017), which demonstrated that ribosomal scores can be used as a measure to estimate the translational efficiency. **Table 1** shows examples from the application of these approaches into model plants. As for proteomics, several factors also contribute to the sequence diversity among the pool of translating mRNAs such as the association of RNA binding proteins to translating mRNAs and translational elongation (Tebaldi et al., 2012; Browning and Bailey-Serres, 2015). Additionally, sequence features such as the length and the GC content of transcripts (Zhao et al., 2017) and the presence or absence of smallORFs, can affect the rate by which mRNAs can be translated (Zhao et al., 2017).

**Table 1:** Summary of the translating mRNAs studies.

Plant species	Condition	Reference
*A. thaliana*	Hypoxia	Branco-Price et al., 2005; Mustroph et al., 2009; Niedojadło et al., 2016
*Zea mays*	Drought stress	Lei et al., 2015
*A. thaliana*	Circadian cycle	Missra et al., 2015
*A. thaliana*	Gibberellin signaling	Ribeiro et al., 2012
*A. thaliana*	Heat stress	Yángüez et al., 2013
*A. thaliana*	Lipid metabolism	Li et al., 2015
*A. thaliana*	Developing flowers	Jiao and Meyerowitz, 2010
*A. thaliana*	Root development	Rajasundaram et al., 2014
*A. thaliana*	Multiple stresses	Palusa and Reddy, 2015
*A. thaliana*	Seed germination	Galland et al., 2014; Bai et al., 2016
*A. thaliana*	Thermal stress	Lukoszek et al., 2016
*Oryza sativa*	Tissue and stress	Zhao et al., 2017
*A. thaliana*	Brassinosteriod signaling	Vragović et al., 2015
*A. thaliana*	Pollen tubes	Lin et al., 2014
*A. thaliana*	Light stress	Gamm et al., 2014

## Translating mRnas and their Roles in Stress Functional Genomics

Transcriptional profiling has played a vital role in understanding the regulation of genes during stress. Also revealed are a wide array of genes involved in stress responses (Kreps et al., 2002; Buitink et al., 2006; Kianianmomeni, 2014; Valliyodan et al., 2014). However, the translating dynamic landscape of these transcriptionally active mRNAs was mostly lacking with only few reports addressing the role of translating mRNAs in stress (Mustroph et al., 2009; Juntawong et al., 2014). Given that the advent of the ribosome- profiling approaches made the identification of translating mRNAs possible we are now in a much better position to determine how transcriptional changes occurring during stress correlate with those of translating mRNAs (Zhao et al., 2017). In the following section, a few specific examples from recent studies will be briefly discussed.

Light plays a key role in the adaptation of plant species to any environment and controls the rate of photosynthesis, which is a source point for energy required for growth. Several stress studies have used light measures and photosynthetic efficiency as a first point to understand plant responses to stress (Dunaeva and Adamska, 2001; Soitamo et al., 2008). Previously, wide arrays of genes have been profiled in response to light stress in model and non-model species and attempts made to reveal the light-based regulation and evolution of C3–C4 biosynthetic cycle (Perduns et al., 2015). Light-regulated responses occur diurnally with alternative light- and dark- phases. Different sets of the transcriptionally active genes have been identified during diurnal shifts (Vialet-Chabrand et al., 2017). Ribosome profiling approaches revealed mRNAs encoding ribulose bisphosphate carboxylase (*RBCS*) and ribulose bisphosphate carboxylase small chain 1A (*RBCS1A*) as major translating mRNAs (Liu et al., 2013; Juntawong et al., 2014). *RBCS* plays an important role in carbon fixation and regulatory role of this enzyme at the level of translation can help understand the minimum cost of translation and translation efficiency for carbon fixation. These observations can be linked to the energy cost of translation, which is defined as the minimum energy required to translate particular mRNAs (Branco-Price et al., 2008) and have also been recently shown in light regulated translating mRNAs (Juntawong and Bailey-Serres, 2012). Missra et al. (2015) observed phase shifts and state transitions of the translating mRNAs with ribosomal proteins and mitochondrial respiration associated translating mRNAs showing peak translation states during night cycles. Notably, central clock mRNAs revealed a wide variation in the abundance of translating mRNAs, with *CCA1*, a clock-associated mRNA, showing phase shifts and light-dark phase regulated ribosomal associations (Missra et al., 2015). These associations reveal the diurnal transition of translating mRNAs involved in the circadian clock (Missra et al., 2015).

Translating mRNAs also showed alterations under various stress conditions (**Table 1**). For instance, under hypoxia (low oxygen stress) only 70% of the cytosolic mRNAs were found to be associated with ribosomes to conserve cellular kinetic energy (Branco-Price et al., 2008; Juntawong and Bailey-Serres, 2012). Among the favored translating mRNAs found under hypoxia were those that either promote the conservation of ATP as a source for cellular energy or facilitate the shift toward anaerobic metabolism (Juntawong and Bailey-Serres, 2012). This observation is consistent with the increase observed in translating mRNAs encoding for anaerobic enzymes (Mustroph et al., 2009), thus providing support for the selective enrichment of mRNAs during physiological changes. Previous observations were further supported by whole genome ribosome foot-printing in hypoxia (Juntawong et al., 2014). Previous reports indicate fewer number of ribosomes associated with up-regulated transcripts under hypoxia thus illustrating the lack of translational initiation as compared to elongation (Juntawong et al., 2014). However, recent studies established the correlation between the transcriptional and translational coordination under drought stress in maize (Lei et al., 2015).

Recent studies highlighting the differences in the pool of translating cytoplasmic and nuclear mRNAs under hypoxia have presented new insights into the selection of translating mRNAs (Niedojadło et al., 2016). For example, no preferential enrichment of translating *ADH1* (*Alcohol Dehydrogenase 1*) mRNAs, a core hypoxia-induced gene, was observed in nucleus among the selectively retained translating mRNAs (Niedojadło et al., 2016). Interestingly, post-aeration (a mechanism to restore the plant from hypoxia-induced stress) revealed selective increase in cytoplasmic mRNAs (Niedojadło et al., 2016). This indicated that cytoplasmic mRNAs come as a first point of contact to the translational machinery during stress recovery while mRNAs, which are not involved in stress responses, are stored pre-dominantly in the nucleus (Niedojadło et al., 2016). Similar patterns of condition-associated selective enrichment of translating mRNAs have been observed by Tiruneh et al. (2013). Juntawong et al. (2013) showed a positive association between cold shock protein (CSP) abundance and the translation of ribosomal mRNAs in *Arabidopsis thaliana*, demonstrating the selective abilities of CSPs as molecular chaperones to selectively load mRNAs.

To understand the processes involved in the regulation of translating mRNAs, genes involved in this process have been identified and functionally characterized using mutant lines. Since translating mRNAs represent the pool of polyA mRNAs associated with ribosomes, it is imperative to highlight the role of PABS, which is a poly-A binding protein that exerts a level of translational control by bringing the 5′ cap and 3′ poly-A tail together (Tiruneh et al., 2013). However, this process is compromised in the poly-(A) binding protein mutant, *pab2 pab8*, as well as in a mutant of a large ribosomal subunit protein, *rpl24b/shortvalve1* (Tiruneh et al., 2013). Comparative assessment of translating mRNAs across these mutants revealed that only one-fifth of the mRNAs showed a highly plastic translational control and the lack of poly-A binding protein mutations has only affected proteins involved in late embryogenesis. However, no significant effect of the *rpl24b* mutation on translating mRNAs was found, suggesting that the pool of translating mRNAs is independent of the *RPL24b* gene.

## Translating mRNA Pools Across Developmental Landscape

The APETALA2 (*AP2*) gene family plays an important role during plant development (Zhao et al., 2007; Wu et al., 2009). Jiao and Meyerowitz (2010) laid the founding work by demonstrating the role of translating mRNAs in flower development by incorporating FLAG-tagged RPL18 (large subunit ribosomal protein L18) protein under the control of either APETALA1 (AP1), APETALA3 (AP3) or AGAMOUS (AG) promoter. Notably, the enrichment of petal and stamen development was seen as enriched in the AP3 domain as compared to the AP1 and AP2 domain. Interestingly, they observed the enrichment of the GO terms specific to chloroplast functions in the AG domains and the abundance and the enrichment of these terms were found to be positively correlated to flower development, suggesting that the chloroplast translating mRNAs play an important role in flower development. Another example highlighting the role of cell specific translating mRNA comes from the *Arabidopsis thaliana* translatome cell-specific mRNA atlas (Mustroph and Bailey-Serres, 2010). Mining of the Arabidopsis translatome cell-specific mRNA atlas revealed mRNAs encoding suberin and cutin biosynthesis proteins showing cell-type specific regulation at the translational level, further suggesting a role for translational regulation in cell determination and differentiation (Mustroph and Bailey-Serres, 2010). Augmenting these previous observations, selective enrichment and distinctiveness of translating mRNAs vary not only across different cell types but also across different tissues. Tissue specific enrichment of translating mRNAs is also supported by recent findings in *Oryza sativa*, revealing a distinct profile of the GC rich translating mRNAs across tissues (Zhao et al., 2017).

The enrichment of the translating mRNAs also showed variations during developmental phases and selective enrichment of the sub-set of steady state mRNAs during plant development (Yamasaki et al., 2015). For example, using 2-D proteomics with a radiolabeled amino acid precursor, namely [35S]-methionine, a higher proportion of translating mRNAs was seen from phase I to phase II, both of which are defined as germination sensu stricto as compared to phase II to phase III (resumption of water uptake) transitions during *Arabidopsis thaliana* seed germination (Galland et al., 2014). Galland et al. (2014) specifically highlighted the role of the nuclear cap-binding complex, which plays an important role in the selective export of nuclear mRNAs. This finding is also in line with those from recent studies suggesting that the nucleus serves as a host for retention of mRNAs and depending on the nature of the stress response, actively selects translated mRNAs and recruits them to the cytoplasmic pool (Niedojadło et al., 2016). Galland et al. (2014) demonstrated the selective mRNA translation during the seed germination using proteomics based approaches, which recently has been re-visited in *Arabidopsis thaliana* illustrating the role of translating mRNAs mainly to two temporal shifts: seed hydration and germination (Bai et al., 2016). Interestingly, they found a significant overlap (25%) with hypoxia regulated translating mRNAs, which might be attributed to low oxygen during the seed germination (Bai et al., 2016). Basbouss-Serhal et al. (2015) demonstrated the involvement of translating mRNAs in seed germination by comparatively analyzing dormant and non-dormant seeds. A correlation could not be established between the transcriptional and translational landscape, except for few genes such as *ACO1, GASA6*, and *HSP70*, leading to the conclusion that seed germination is mainly translationally controlled. A closer look at the functional categories using GO analysis demonstrated specific enrichment of GO categories in non-dormant and dormant seeds specifically highlights the role of redox and lipid metabolism associated genes in dormancy maintenance (Basbouss-Serhal et al., 2015).

Translational regulation through the regulation of translating mRNAs also plays an important role in sexual reproduction, specifically within pollen tube growth. Transcriptomics based approaches have highlighted the role of *POP2*, which plays an important role in pollen tube growth (Palanivelu et al., 2003). The role of LURE peptides, which are defensin-like peptides secreted from synergids has been widely elucidated as signaling components (Kanaoka and Higashiyama, 2015). However, the detection of translating mRNAs has been lacking until the studies of Lin et al. (2014), which used LAT52: HF-RPL18 transgenic Arabidopsis expressing the ribosomal protein L18 (RPL18) tagged with a His6-FLAG and driven by pollen specific promoter (ProLAT52) (Twell et al., 1989). A comparative analysis of the *in vivo* and *in vitro* pollen tubes showed 41 specific transcripts that were enriched in *in vivo* pollen tubes, including *IV6* (xyloglucan endotransglucosylase/hydrolase), *IV4* (putative glutathione transferase) and *IV2* (putative methylesterase), which are involved in micropylar guidance. Lin et al. (2014) also highlighted the difference in the pool of translating mRNAs as compared to the previously transcribed mRNAs suggesting the difference in the steady state population of mRNAs and ribosome associated mRNAs (Lin et al., 2014).

Hormonal regulation plays an important part in plant growth (Fridman et al., 2014). Plant patterning and architecture is a widely studied developmental process with most studies focusing on spatio-temporal regulation of shoot apical meristem (Gendron et al., 2012). Hormonal regulation of translating mRNAs dates back to the first study by Jiao and Meyerowitz (2010). These authors, using AP1, AP2, and AP3 domain specific pool of translating mRNAs, highlighted the role of several hormones such ethylene, jasmonic acid, brassinosteriods, cytokinins, and gibberellins in the regulation of translating mRNAs. Specifically, they observed that genes with in AP3 domains showed pattern of up- and down-regulation at specific stages of flower development in response to gibberellins and jasmonic acid. Notably, they observed these phytohormones regulate the flower development by down-regulating the specific genes in the AP3 domain (Jiao and Meyerowitz, 2010). Ethylene plays a central role in plant development and most importantly its perception to the stomatal opening and activating the stress perception in plants. Among the most widely characterized ethylene pathways, *EIN2* (ETHYLENE INSENSITIVE 2), which plays a key role in the perception and signaling of the response from the endoplasmic reticulum to nucleus (Zheng and Zhu, 2016) has been shown to be under the translational control previously using the ribosome profiling methods. Interestingly, in parallel to the EIN2, non-sense mediated decay proteins UPFs also act synergistically to control the translational control of *EIN2* (Merchante et al., 2015). It has been further demonstrated that the translational control of the *EBF3*, which is a critical component of the master ethylene pathway is under the translational control of the EIN2, UBFs and 3′ long UTRs of EBFs (Merchante et al., 2015).

Ribeiro et al. (2012), using the FLAG-epitope tagged ribosomal protein L18 (FLAG-RPL18), demonstrated the role of gibberellins (GAs) in modulating the pool of translating mRNAs in *Arabidopsis thaliana* shoots. Translating mRNA profiling revealed the feedback regulation of GA biosynthetic genes, demonstrating the correlation between the carbon availability and growth. The role of brassinosteroids has been widely elucidated in regulating root and shoot development (Fridman et al., 2014). Recently *BZR1*, a brassinosteriod specific transcription factor, has been shown to regulate the expression of transcripts involved in development (Jaillais and Vert, 2016). A recent investigation highlights the role of *BZR1* in suppressing the cup-shaped cotyledon (CUC) gene, which regulates the morphogenesis processes taking place in the shoot apical meristem (Gendron et al., 2012). Translating mRNA-profiling approaches revealed tissue specific regulation of BR (Vragović et al., 2015). Interestingly, contrasting patterns of gene expression were observed, with epidermal cells inducing the cell division as a signal from BR by stimulating auxin gene expression and stele suppresses the epidermal induction (Vragović et al., 2015) resulting in coordinated growth and meristem size determination. Perception and involvement of auxin in TOR signaling pathway has been first elucidated by uncovering the translational control of the up-stream open reading frame (uORF), which depends on the translational elongation factor eIF3h (Schepetilnikov et al., 2013). To delineate the functional association TOR inhibitor Torin-1 was used, which in case of non-inhibition activity recruits the SK6K1 to polysomes for phosphorylation, whereas in the presence of the Torin-1 auxin promotes the SK61 dissociation and functional association of the TOR to polysomes thus functionally eludicating the TOR pathway, which plays a key role in response to hormones and nutrients (Schepetilnikov et al., 2013).

## Features Affecting Selective Recruitment of Translating mRNAs

Translating mRNA pools have been widely studied to understand sequence features that allow for the selective association of translating mRNAs with ribosomes under specified conditions (Zhao et al., 2017). Among the features that have been widely correlated with translating mRNA are the GC content, minimal free energy and uORFs, which act as a check point for translating mRNAs (Lei et al., 2015). For example, a recent study by Zhao et al. (2017) indicated the selective enrichment of GC rich and short coding sequences with translating mRNAs across tissues. Similar features have been observed during stress and development, suggesting that plant translating mRNAs represent the minimum energy cost budget defined as the minimal energy required for subsequent elongation and termination of translating mRNA (Juntawong et al., 2013; Basbouss-Serhal et al., 2015). It is interesting to see that minimal free energy is one of the factors that also controls the population of translating mRNAs and their subsequent association with ribosomes (Lei et al., 2015). Minimal free energy affects RNA folding and has been previously widely linked to the ribosomal rates, which is defined as the rate of the association of the ribosome to the corresponding mRNAs. Recently, this has been addressed using the ribosome drafting technique, which typically links the accelerated rate of ribosome binding to mRNAs as compared to the canonical rate of mRNA folding (Borujeni and Salis, 2016). However, whether ribosomes drafting occurs for transcripts that accelerate to populate with the ribosomes under defined abiotic or biotic stress conditions in plants has not yet been established.

In addition to sequence features, recent investigations by Lukoszek et al. (2016) have demonstrated the role of mRNA secondary structures which influence the association of translating mRNAs with ribosomes. Secondary mRNA structures can have a direct influence on their folding energy as well. Lukoszek et al. (2016) estimated the folding energy of profiled mRNA and found that up-regulated translating mRNAs have relatively higher folding energy up-stream of the start codon. Interestingly, this scenario has not been observed in the case of down-regulated translating mRNAs, suggesting that the up-stream enhancement of the folding energy is a feature associated with rapidly translating mRNAs to increase the ribosomal occupancy at a given time point. Previous studies have shown that mRNAs with stable structures encode proteins that are more compact and mRNA length acts as a determinant of the folding energy (Faure et al., 2016). Several studies have indicated toward the selective recruitment of shorter transcripts with high GC content to ribosomes (Liu et al., 2013; Zhao et al., 2017). However, the correlation of GC richness with codon usage, a measure that represents the usage of codons in synonymous sites, needs to be also taken into account as the biased and preferential usage of codons varies from one species to another. Interestingly, Bai et al. (2016) observed a positive correlation between translating mRNAs and GC content at the third synonymous sites in codons, providing the basis of selective enrichment of the GC rich transcripts for frequent association to ribosomes. In addition to the aforementioned sequence features, translating mRNAs such as the ones pertaining to seed germination have been recently shown to have enriched motifs (Motif3c) (Bai et al., 2016). It is likely that the presence of this motif allows enhanced translational initiation (Bai et al., 2016).

## Translating mRNAs and Splicing Diversity

Protein diversity and the evolution of protein diversity through the mechanism termed alternative splicing (AS) has been widely studied in plants (for a review see Reddy et al., 2013). Plants show a high proportion of splicing diversity with as much as 60% of the *Arabidopsis thaliana* proteome is resulted from alternatively spliced transcripts (Reddy et al., 2013). Juntawong et al. (2014) provided the first evidence of translating spliced mRNAs, revealing a link between alternative splicing and the ribosomal association of spliced transcripts. The preferential ribosomal association of the most abundantly spliced gene family (serine/arginine-rich (SR) proteins) has recently been shown (Palusa and Reddy, 2015). The non-small nuclear ribonucleoprotein (snRNP) spliceosomal protein family shows a differential recruitment of splice variants during development and in response to heat and cold stress (Palusa and Reddy, 2015). There are 100 distinct splice variants from 14 SR genes in this family (Reddy et al., 2013). However, ribosomal association seems to be affecting only three *SR* genes (*SR30, SR34a*, and *SR34b*) and their splice variants (Palusa and Reddy, 2015). Intron retention, a dominant form of splicing variation in plants (Mastrangelo et al., 2012; Min et al., 2015) has been shown to regulate the preferential recruitment of these splice variants as translating mRNAs (Palusa and Reddy, 2015). Interestingly in *Oryza sativa*, features of translating mRNAs revealed fewer association of transcripts with retained introns to ribosomes (Zhao et al., 2017), which is in line with the translating mRNAs observed in seed germination (Bai et al., 2016). Together, these findings suggest that exploring the association between intron splicing and translating mRNAs may be able to establish the role of splicing machinery at translational level. This in turn will help unravel how splicing machinery may alter the translational output in species-specific alternative splicing and may reveal differential pools of spliced translating mRNAs associated with development and abiotic stress in plants.

## Translating mRNAs and Polyploidy

It is now widely accepted that the majority of extant plants are currently polyploid (neopolyploid) or were in a state of polyploidy at some point in their evolutionary history (paleopolyploid) (Wood et al., 2009). A subsequent effect of whole genome duplication or ploidy-induced chromosome doubling is the abundance of in-paralogs with respect to orthologous genes. Following polyploidization, global transcriptional patterns shift dramatically relative to patterns observed in progenitor species. Following this initial state of flux, polyploids move toward functional diploidization. During this evolutionary process, redundant genes are silenced or lost (Schnable et al., 2011; Sehrish et al., 2014) and the patterns of homoeolog expression bias and expression dominance are established (Feldman and Levy, 2009). Therefore, polyploidy-associated phenomena should be taken into consideration when studying translating mRNAs within polyploid crop species.

Previous studies by Tiruneh et al. (2013) revealed differences in translating paralogous mRNAs encoding ribosomal proteins; however, whether the correlation of this observed translational state to the *rpl24b* mutation is yet to be established. Interestingly, a difference in the association of paralogous translating mRNAs with ribosomes was seen in *Glycine dolichocarpa* (∼100 MYA allotetraploid), revealing wide variations in one-quarter of the translating mRNAs, with categories mostly involved in photosynthesis (Coate et al., 2014). Specifically, Coate et al. (2014) indicated that translational shifts might be possible in polyploid genomes, and can cause expression shifts in whole chromosome homoeologs. Transcriptional response in hexaploid wheat genome indicates a sub-genome bias toward the transcriptional response to biotic stresses indicating a preferential expression of defense related genes from B and D sub-genomes (Powell et al., 2017). However, it is yet to be ascertained whether the homoeolog expression divergence observed occurred at the translating mRNA level. However, translating mRNA regulations represent association with homoeologs retained with after paleopolyploid event and provide a proof of concept for further exploration of links between the role of homoeologs and their subsequent association and divergence with ribosomes.

## Conclusion and Future Directions

Recent profiling of the transcriptional landscape in crop and model plants has produced numerous insights that can be used to potentially enhance crop productivity by aiding selection of genotypes resilient to stresses. However, a thorough understanding of post-transcriptional changes and in particular translating changes is still lacking. Recently developed techniques such as ribosomal profiling has started revealing potential roles of translating mRNAs in stress responses (Browning and Bailey-Serres, 2015), which will be the next major leap forward to accelerate the improvement of stress tolerance in diverse crop species.

## Author Contributions

GS conceived and drafted the MS. JP and KK provided input and revisions to the manuscript.

## Conflict of Interest Statement

The authors declare that the research was conducted in the absence of any commercial or financial relationships that could be construed as a potential conflict of interest.
